# Curing Pressure Impacts on Strength, Drying Deterioration and Pore Structure of Two-Component Cement–Sodium Silicate Grout

**DOI:** 10.3390/ma19153326

**Published:** 2026-08-05

**Authors:** Wenxue Wang, Lu Guo, Yuan Fang, Haolin Gong, Yang Li, Kun Zhang, Jian Chang, Jiawei Liu, Shuli Zhao

**Affiliations:** 1 Henan Key Laboratory of Geomechanics and Structure Engineering, North China University of Water Resources and Electric Power, Zhengzhou 450045, China; 2 Henan Provincial Hydrology and Water Resources Monitoring and Forecasting Center, Zhengzhou 450003, China; 3 China State Construction Engineering Corporation Sixth Bureau Eighth Construction Co., Ltd., Guangzhou 510800, China; 4 Henan Key Laboratory of Engineering Materials and Hydraulic Structures, School of Intelligent Construction and Transportation Engineering, Henan University of Urban Construction, Pingdingshan 467036, China

**Keywords:** cement–sodium silicate binary grout, curing pressure, unconfined compressive strength, drying-induced deterioration, microstructure, pore characteristics

## Abstract

Cement–sodium silicate binary grout is widely used for water sealing and stratum reinforcement in deep underground engineering. Curing pressure profoundly affects the mechanical performance and microstructure of the hardened grout, whereas its pressure-dependent mechanical responses remain insufficiently understood. This study comprehensively investigates the grout’s workability, mechanical properties and water-loss degradation characteristics, combining scanning electron microscopy (SEM) and mercury intrusion porosimetry (MIP) for microscopic analysis. The results show that the combined addition of 3% sodium bentonite and 1% polycarboxylate superplasticizer effectively mitigates slurry bleeding. An increased cement-sodium silicate volume ratio (C/S) improves fluidity and gel time, and C/S ratios of 0.4, 0.5 and 0.6 were adopted for mechanical tests. Under ambient conditions, grout compressive strength increases with curing age but declines at higher C/S ratios. Curing pressure presents a non-monotonic influence on strength: the strength reaches a minimum at 1.0 MPa and partially recovers at 2.0 MPa, which may result from the competition between pore compaction and hydration gel network damage. Water-loss-induced strength degradation undergoes three typical stages. Grout with lower C/S ratios cured under higher pressure possesses better crack resistance and residual strength. This study clarifies the pressure-adapted mechanism of the grout, providing guidance for its optimal proportioning and application in high-pressure underground engineering.

## 1. Introduction

Cement-based binary grout has been widely used in underground engineering, tunnel water sealing, foundation reinforcement, and submarine construction because of its rapid gelation, favorable sealing capacity, and reliable mechanical performance [1,2,3]. The engineering behavior of hardened grout is jointly controlled by material composition, ambient temperature, water content, and external loading conditions [4,5]. Among these factors, curing pressure, which may originate from overburden stress, seepage pressure, and in situ stress, plays an important role in strength development, deformation behavior, and internal structural evolution. Therefore, clarifying the effect of curing pressure on cement-based binary grout is essential for mix design and construction-parameter optimization in grouting engineering.

Laboratory and field investigations consistently confirm that curing pressure exerts a reinforcing effect on cement-stabilized geomaterials and single-component cement grouts. Within low-to-medium stress ranges, increased curing confining pressure can enhance unconfined compressive strength (UCS), yield strength, and stiffness in cement-treated soils and grout stones. 

He et al. [6] reported that a curing stress of 100 kPa increased the yield stress of cement-modified dredged sediment by two to four times, with a stronger effect at lower cement dosages. For high-water-content cement-stabilized clay, Zhang et al. [7] found that an 80 kPa curing pressure increased UCS by up to 536% and proposed a dual strengthening mechanism involving physical consolidation and residual self-stress induced by cementitious bonds. At medium-to-high pressures of 2–10 MPa, the strength growth of cement-based grout no longer followed a simple linear trend but was better described by a quadratic relationship [8]. Other studies have examined cement-based materials under high and ultra-high curing pressures, showing that the effects of pressure may depend strongly on temperature, material composition, and hydration stage [9,10,11,12,13,14].

From the perspective of yield behavior and compressibility, Consoli et al. [15] established reliable linear relationships among the incremental yield stress, initial bulk modulus, and UCS of artificially cemented silty sand cured under different confining stresses. They noted that the lower curing void ratio induced by higher pressure could amplify the cementation effect. Rotta et al. [16] further demonstrated that high curing pressure significantly improved the bulk modulus and isotropic yield stress of cemented soil by reducing the void ratio. Suzuki et al. [17] compared cement-treated soil subjected to isotropic and one-dimensional consolidation and showed that both peak and residual shear strengths increased with consolidation pressure during curing, although the strength growth rate differed significantly under different stress states. Microscopic studies have provided further insight into the mechanisms of strength variation under curing pressure. External pressure may accelerate the early hydration reaction of cement by inducing microcracks inside cement particles and promoting water infiltration [18]. In addition, curing pressure can rearrange solid particles, reduce overall porosity, and refine the pore-size distribution, thereby forming a denser internal structure [5,19]. Qiu et al. [5] also pointed out that the strengthening effect of curing pressure gradually weakened with curing time, and that excessive cement content might even lead to premature failure of consolidated bodies under high pressure. Lin and Meyer [20] developed a hydration-kinetics model incorporating pressure and temperature, which provides a theoretical basis for analyzing cement hydration under complex stress conditions.

Existing investigations on the mechanical responses of cementitious materials under curing confining pressure predominantly target single-component cement grout, cement-stabilized geomaterials, and oil-well cement, with nearly all works reaching a consistent conclusion that compressive strength increases monotonically with elevated confining pressure. However, such established conclusions are not directly transferable to cement–sodium silicate binary grout. Distinct from monophasic cement matrices, the blending of cement slurry and sodium silicate solution initiates instantaneous gelation accompanied by synchronous rapid hydration and multiphase mineral phase transformation. These unique coupled physicochemical reactions reconstruct the pressure–strength correlation and microstructural evolution pathways, which fundamentally differ from those of conventional single-component cementitious materials. This constitutes a prominent research gap: dedicated experimental efforts are absent to identify whether binary grout obeys the monotonic strength trend reported in prior literature, and the pressure-governed microstructural mechanisms remain unelucidated. 

Accordingly, this study explicitly proposes its scientific hypothesis: the compressive strength of hardened cement–sodium silicate binary grout exhibits non-monotonic variations with increasing curing confining pressure. Specifically, low-to-medium confining pressure disrupts the interconnected early hydration gel network and generates microstructural defects, leading to strength degradation, whereas high confining pressure densifies the matrix and rearranges solid particles to partially restore load-bearing capacity. In practical underground grouting engineering, in situ geostress and water pressure continuously impose confining curing loads on grouted rock and soil masses. The aforementioned research gap hinders accurate performance evaluation of grouted stone and rational optimization of critical construction parameters, including mix proportion, grouting pressure and curing duration. To bridge this theoretical deficiency and validate the proposed hypothesis, the present work systematically investigates the pressure-dependent strength evolution, drying degradation characteristics and inherent microstructural mechanisms of binary grout subjected to diverse curing confining pressures. The obtained findings advance the theoretical framework of pressure-cured cement-based grouting composites and offer theoretical support for field implementation of binary grouting under high in situ stress environments.

## 2. Materials and Methods for Performance Tests of Binary Grout

### 2.1. Test Materials

The test materials included P.O 42.5R ordinary Portland cement (Henan Tianrui Group Co., Ltd., Zhengzhou, China), sodium silicate solution (Jiashan Yourui Refractory Materials Co., Ltd., Jiaxing, China), sodium bentonite (Xinyang Industrial City Tongchuang Bentonite Factory, Xinyang, China) as a suspending agent, polycarboxylate superplasticizer (Henan Kegong Building Materials Co., Ltd., Zhengzhou, China), and laboratory tap water. The sodium silicate solution had a modulus of 3.3, and the Baumé degree of the stock solution was 39 °Bé, with a density of 1.366 g/cm^3^. The swelling coefficient of sodium bentonite was 23.48 mL/g, and its montmorillonite content was not less than 95%. The polycarboxylate superplasticizer had a solid content of 10.8% and a water-reducing rate of 19%.

The binary grout consisted of cement slurry (Component A) and sodium silicate solution (Component B). Component A was prepared by mixing the raw materials according to the designed water-cement ratio and admixture dosages, whereas Component B was diluted to the required sodium silicate concentration. The cement-sodium silicate grout was prepared by rapidly mixing Components A and B at a prescribed cement slurry-to-sodium silicate solution volume ratio, hereafter referred to as the C/S volume ratio. To ensure comparability, the water-cement ratio of Component A was fixed at 0.8, the bentonite dosage at 3%, and the superplasticizer dosage at 1% in all subsequent tests unless otherwise specified. All parameter tests described below were repeated more than three times for each group, the test results were deemed valid if the relative error among the three measurements was less than 10%; otherwise, the tests were repeated. The average value of three sets of data with consistent test results was adopted as the final measurement result. All experiments were carried out in the Henan Key Laboratory of Geomechanics and Structure Engineering, with an indoor relative humidity of 43~50%. The detailed experimental design and corresponding test results are analyzed in the following sections.

### 2.2. Test Scheme

The working-performance tests included measurements of bleeding rate, fluidity, and gel time.

**Figure 1:**
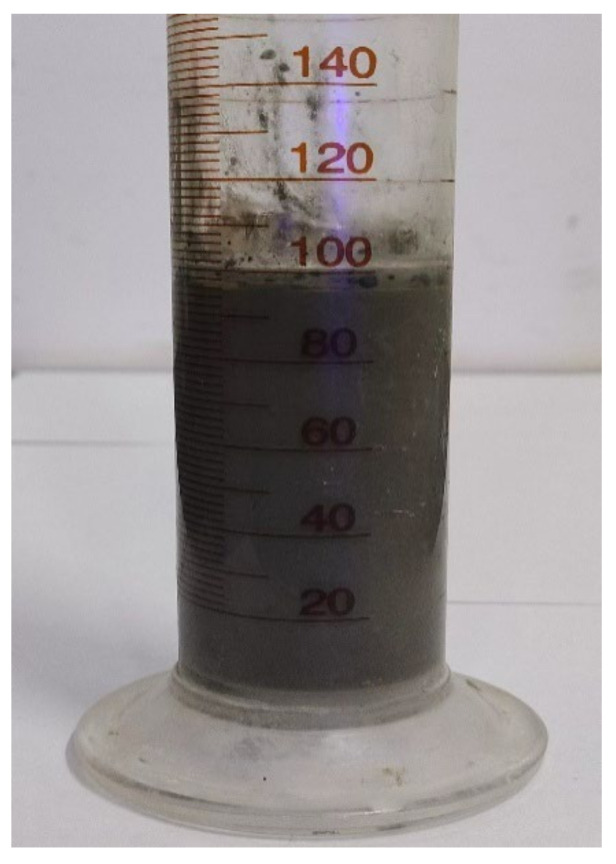
Bleeding-rate test.

**Figure 2:**
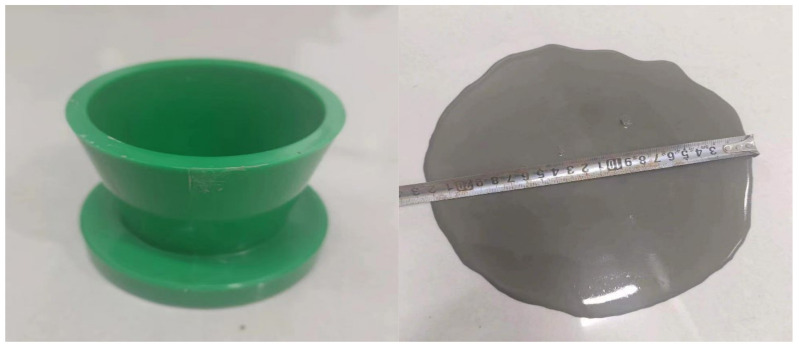
Fluidity test.

(1) The bleeding rate was measured using the graduated-cylinder static settlement method (Figure 1). A graduated cylinder with a precision of 2 mL was used in this test. It was defined as the ratio of the volume of the upper clear water to the total grout volume after the system reached a stable state. Component A with a water-cement ratio of 0.8 was used, and the bleeding rate was measured under different dosages of the suspending agent and superplasticizer.
(2) Fluidity was tested using a truncated-cone mold (Figure 2). The freshly mixed binary grout was poured into the mold until the liquid level was flush with the top edge. The mold was then lifted vertically and smoothly, allowing the grout to spread freely until flow ceased. The spread diameter was then measured. The steel ruler has a measurement precision of 1 mm.

Binary grouts with three sodium silicate concentrations (39 °Bé, 36 °Bé, and 32 °Bé) and C/S volume ratios of 0.4, 0.5, 0.6, and 0.7 were tested. Component A was prepared with a water–cement ratio of 0.8, a sodium bentonite dosage of 3%, and a superplasticizer dosage of 1%. Because the binary grout set rapidly at room temperature, timing was terminated when the spread grout had fully solidified.

**Figure 3:**
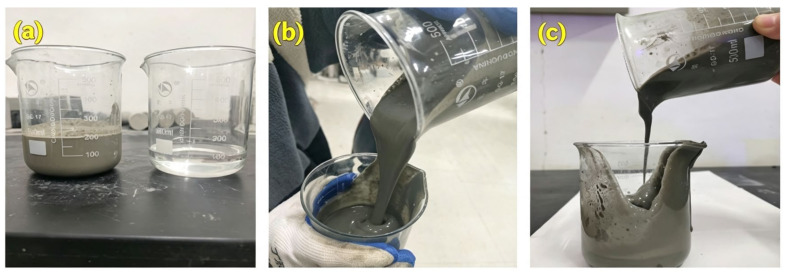
Gel-time test.

(3) Gel time was determined using the cup-inversion method. Test procedure for gel time of double-liquid grout as shown in Figure 3: (a) Pre-prepared cement slurry (liquid A) and sodium silicate solution (liquid B) are separately filled in two beakers according to the test mixing ratio; (b) Quickly pour liquid B into liquid A and start timing, then pour the mixed grout back and forth between two beakers to maintain fluidity; (c) Stop timing once the mixed double-liquid grout loses flowability, and the recorded time value is the gel time of the grout. The elapsed time was recorded when the grout lost flowability, the timer used in the test had a precision of 1 s.
  The gel time of the binary grout was measured at C/S volume ratios ranging from 0.3 to 0.7 using sodium silicate concentrations of 32 °Bé and 39 °Bé.

### 2.3. Analysis of Test Results for Grout Properties

#### 2.3.1. Bleeding Rate

**Figure 4:**
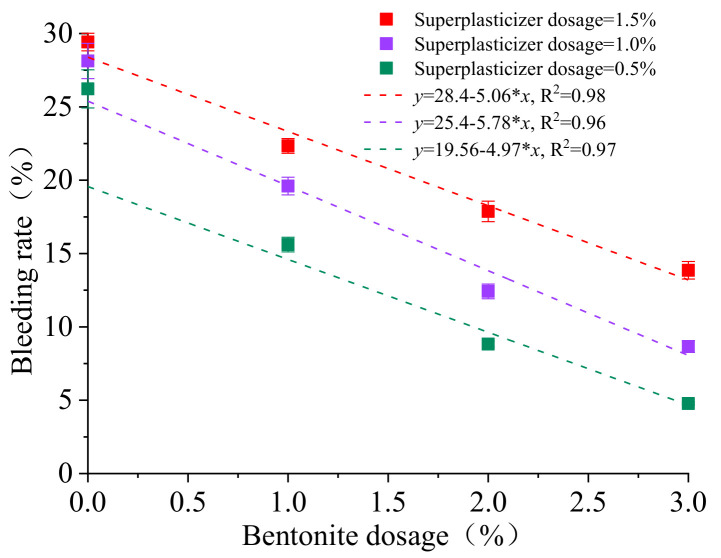
Bleeding rate of Component A under different bentonite dosages.

At a fixed water–cement ratio of 0.8, the sodium bentonite dosage had a pronounced inhibitory effect on the bleeding rate of the cement slurry (Figure 4). As the bentonite dosage increased from 0% to 3%, the bleeding rate decreased markedly for all superplasticizer dosages, with a reduction exceeding 60%. A clear negative linear correlation was observed between the bleeding rate and bentonite dosage, indicating that bentonite effectively improved the suspension stability of the grout.

**Figure 5:**
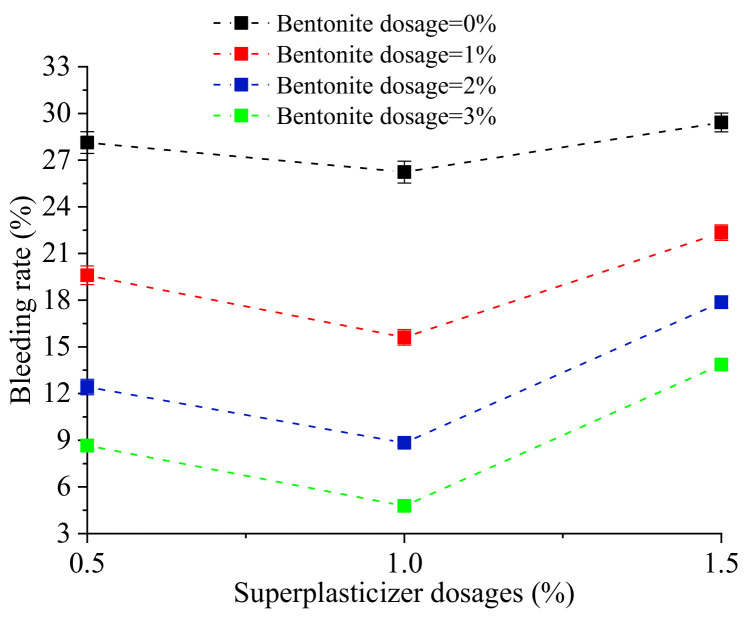
Bleeding rate of Component A under different superplasticizer dosages.

At the same bentonite dosage, the bleeding rate first decreased and then increased as the superplasticizer dosage increased (Figure 5). For each bentonite dosage, the minimum bleeding rate occurred at a superplasticizer dosage of 1%. Further increasing the superplasticizer dosage caused excessive dispersion, reduced grout stability, and led to a rebound in the bleeding rate. Accordingly, the recommended dosages of bentonite and superplasticizer were determined to be 3% and 1%, respectively. All of the bleeding rate test results are shown in Table 1.

#### 2.3.2. Fluidity

**Figure 6:**
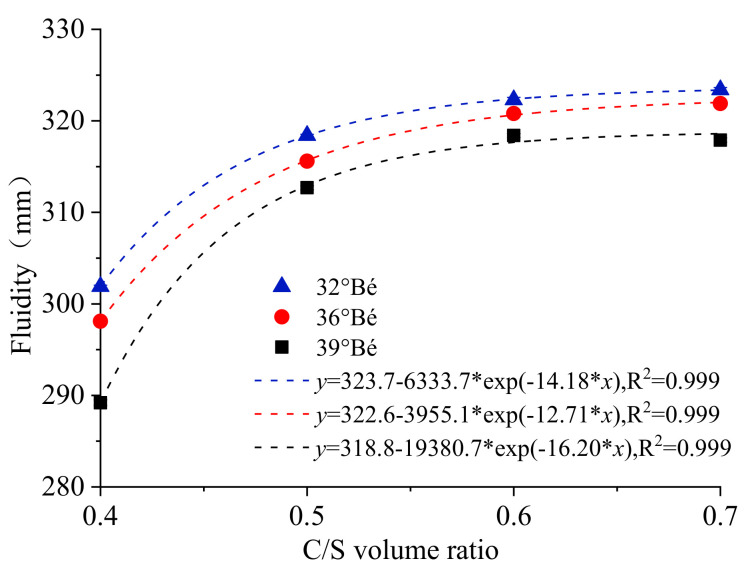
Effect of C/S volume ratio on the fluidity of binary grout.

Figure 6 and Table 2 show the effect of the C/S volume ratio on the fluidity of the binary grout. Within the tested C/S volume ratio range of 0.4–0.7, the fluidity of grouts prepared with the three sodium silicate concentrations (32 °Bé, 36 °Bé, and 39 °Bé) increased gradually with an increasing C/S volume ratio. This indicates that the C/S volume ratio is one of the dominant factors controlling grout fluidity. At a given C/S volume ratio, the fluidity followed the order 32 °Bé > 36 °Bé > 39 °Bé, demonstrating that increasing the sodium silicate concentration reduced grout fluidity.

#### 2.3.3. Gel Time

**Figure 7:**
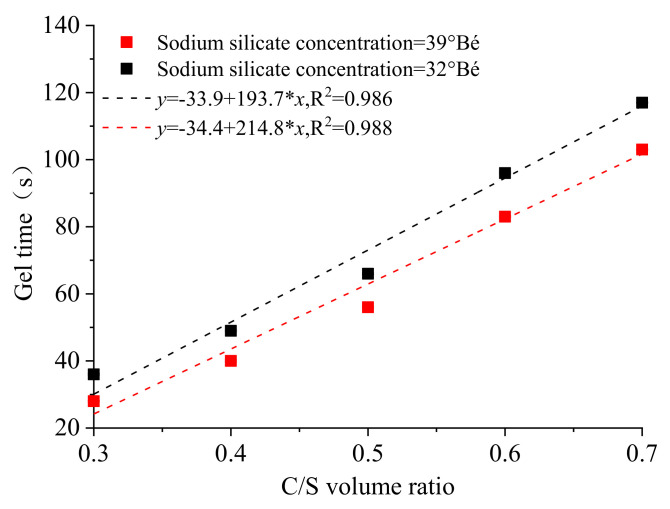
Effect of C/S volume ratio on gel time.

As shown in Figure 7 and Table 3, at sodium silicate concentrations of 32 °Bé and 39 °Bé, the gel time increased monotonically with the C/S volume ratio. At the same C/S volume ratio, the grout prepared with te 32 °Bé sodium silicate exhibited a longer gel time than that prepared with the 39 °Bé sodium silicate. This result indicates that increasing the sodium silicate concentration accelerates gelation and shortens the gel time of the binary grout.

## 3. Experimental Study on the Mechanical Properties of Hardened Binary Grout

Based on the fluidity and gel-time results and practical engineering requirements, C/S volume ratios of 0.4, 0.5, and 0.6 were selected for subsequent mechanical tests. A C/S ratio of 0.3 produced excessively rapid gelation, which may cause pipeline blockage during grouting, whereas a C/S volume ratio of 0.7 resulted in a prolonged gel time and might increase the risk of washout before consolidation in high-water-pressure environments.

Component B was prepared using sodium silicate with a modulus of 3.3 and a concentration of 39 °Bé. Component A was prepared with a water–cement ratio of 0.8, 3% sodium bentonite, and 1% polycarboxylate superplasticizer. All tests were conducted at room temperature (20 °C).

**Figure 8:**
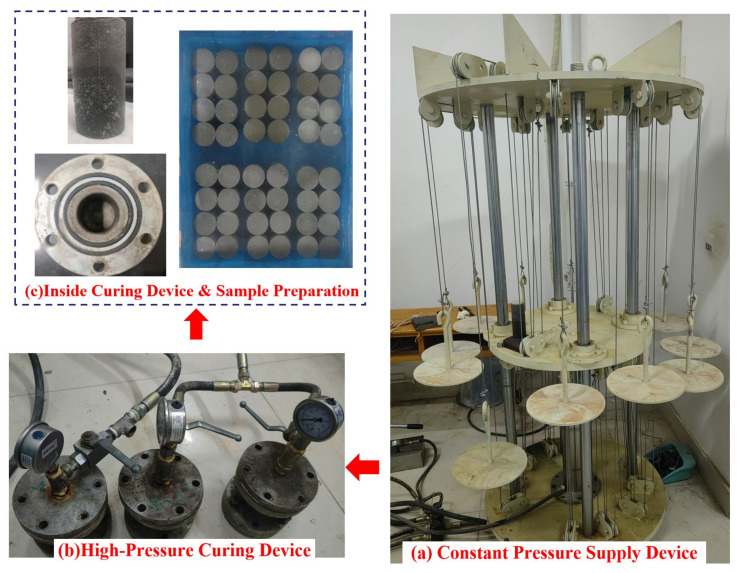
Device for applying different curing pressures and prepared specimens.

Figure 8 Schematic photograph of the constant-pressure curing experimental setup: Figure 8a Constant Pressure Supply Device, which provides a stable vertical curing load for multiple specimens simultaneously; Figure 8b High-Pressure Curing Device equipped with pressure gauges and control valves to achieve a sealed high-pressure curing environment; Figure 8c Inside Curing Device and Sample Preparation, including cylindrical hardened slurry specimens, sealed flange end caps, and forming molds for casting cylindrical samples. The binary grout was poured into the curing device to prepare cylindrical specimens with a diameter of 50 mm and a height of 100 mm. After the device was sealed, the designated curing pressure was applied. The curing pressures were 0, 0.5, 1.0, and 2.0 MPa. After the preset pressurized curing duration, the specimens were removed and cured in water until the target age before mechanical testing.

### 3.1. Effects of C/S Volume Ratio and Curing Age on Compressive Strength

**Figure 9:**
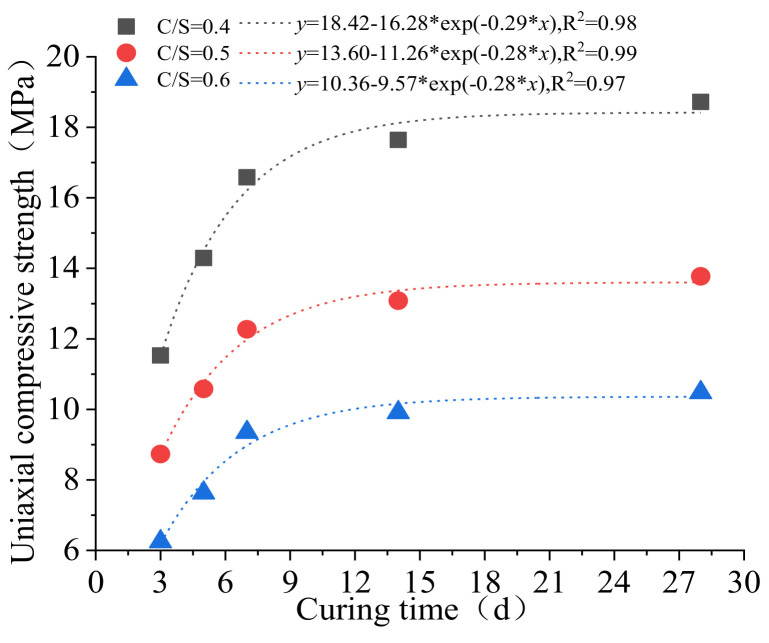
Relationship between compressive strength and curing age of hardened binary grout.

To analyze the strength evolution of hardened binary grout with curing age, UCS tests were conducted on specimens with C/S volume ratios of 0.4, 0.5, and 0.6 under zero curing pressure at different curing ages. The results are shown in Figure 9.

As shown in Figure 9, the compressive strength of specimens with different C/S volume ratios increased with curing age and gradually stabilized. Strength development was well described by the exponential function y = a − b exp(−kx), and all coefficients of determination (R^2^) exceeded 0.97. Strength increased rapidly during the early curing stage (0–14 d). After 14 d, hydration slowed and the strength-gain rate decreased. The compressive strength at 7 days accounted for approximately 89% of the 28-day reference strength, whereas the 14-day compressive strength reached nearly 95% of the 28-day value.

Jiang et al. [21] investigated cement-modified slurry (CMS) cured for 7–180 days with 5–25% cement dosages. The unconfined compressive strength (UCS) of CMS grew rapidly before 90 d and gradually plateaued afterwards; the 180 d strength reached 2.25–2.8 times the 7 d value. The UCS presented a linear logarithmic correlation with curing age as *q* = *A*ln(*t*) + *B*, where coefficients A and B are quadratic functions of cement content. The unified prediction formula achieved a correlation coefficient of 0.991, and 20% cement was identified as the optimal dosage with slowed marginal strength growth above this threshold. Lian et al. [22] proposed a hydration-controlled strength model for pure and blended cement paste cured for 3–90 d. Strength gain is governed by a temperature-corrected equivalent hydration degree following an exponential function. The unified model introduces density-corrected effective water-cement ratio, yielding a fitting R^2^ = 0.96. Low w/c ratios sustain steady strength improvement up to 90 d without obvious plateau; mineral admixtures only play physical filling roles before 28 d without altering the core time-strength trend.

Therefore, it can be concluded that compressive strength rises rapidly in the early curing stage, then the growth rate declines and gradually approaches a stable ultimate strength. Both logarithmic and exponential fitting models well describe this trend with high correlation coefficients, verifying the universal time-dependent strength growth characteristic of cementitious materials.

At the same curing age, UCS followed the order C/S = 0.4 > C/S = 0.5 > C/S = 0.6. This indicates that lowering the C/S volume ratio improved compressive strength, whereas the C/S volume ratio mainly controlled the ultimate strength rather than the rate of strength development.

### 3.2. Influence of Curing Pressure on Unconfined Compressive Strength

#### 3.2.1. Effect of Pressurized Curing Duration on Strength

Binary grout is an early-strength material, and its strength may depend on the duration of pressurized curing. To evaluate this effect, specimens with a C/S volume ratio of 0.4 were cured under pressures of 0.5, 1.0, and 2.0 MPa for 1, 3, 5, and 7 d, respectively. Specimens cured under pressure for less than 7 d were subsequently transferred to water under atmospheric pressure until the total curing age reached 7 d. UCS tests were then conducted to determine the influence of pressurized curing duration on the 7 d strength.

**Figure 10:**
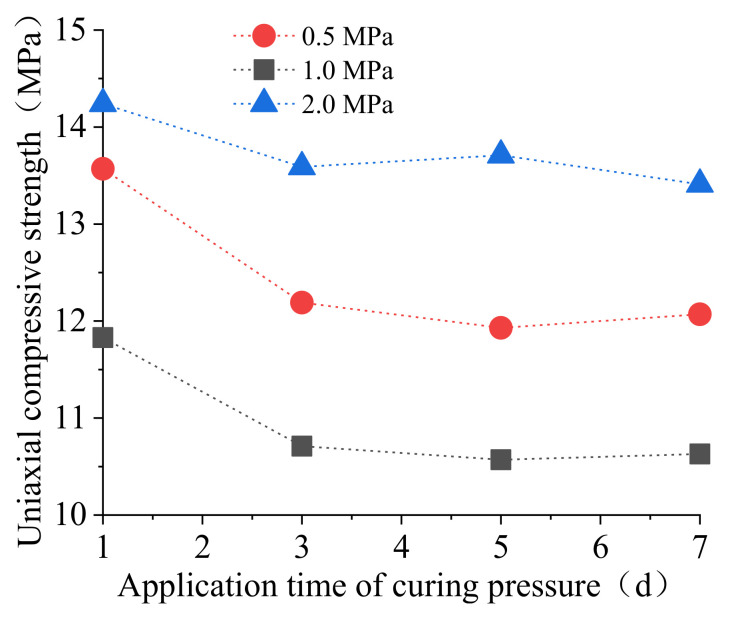
Effect of pressurized curing duration on unconfined compressive strength.

The effect of pressurized curing duration on strength was most pronounced during the early curing stage (Figure 10). Under all three pressures, strength decreased rapidly at first and then gradually stabilized as the pressurized curing duration increased. When the duration increased from 1 to 3 d, the strength reduction rates at 0.5, 1.0, and 2.0 MPa were 10.17%, 8.47%, and 4.56%, respectively. In contrast, strength changed only slightly from 3 to 7 d. These results indicate that curing pressure mainly affected the formation and connectivity of early hydration products; once the internal structure became relatively stable, the effect of pressurized curing duration weakened.

At the same pressurized curing duration, specimens cured at 2.0 MPa exhibited the highest compressive strength, followed by those cured at 0.5 MPa, whereas the lowest strength was obtained at 1.0 MPa.

The variation in uniaxial compressive strength of binary grout with pressurized curing duration may be tentatively attributed to the competing effects of pressure densification and microstructure deterioration. For specimens subjected to 1–3 d of pressurized curing, it is speculated that the grout matrix remains plastic with an incompletely developed C-S-H gel skeleton. Sustained pressure might drive the migration of internal free water and unconsolidated gel, potentially forming irreversible interconnected microcracks and enlarged pores within the matrix. Subsequent atmospheric water curing can only generate limited secondary hydration products, which presumably cannot completely repair these penetrating fractures, possibly resulting in an obvious decline in 7 d compressive strength. When pressurized curing extends to 3–7 d, most hydration reactions are likely completed, forming a rigid solid skeleton. Prolonged pressure can hardly induce new structural defects. Meanwhile, residual unhydrated minerals may continuously produce minor hydration gels to fill tiny pores, counteracting mild structural degradation and stabilizing the overall strength. Under the same pressurization duration, the 2.0 MPa groups show the highest strength, which is presumed to arise from dominant compaction benefits outweighing microcrack damage. Conversely, 1.0 MPa specimens deliver the lowest strength; this medium pressure may bring weak compaction while facilitating severe microcrack propagation, where the destructive influence of pressurized curing on the initially articulated hydration skeleton may outweigh the strength increment induced by pressure densification.

#### 3.2.2. Effect of Curing Pressure on Consolidated Strength

**Figure 11:**
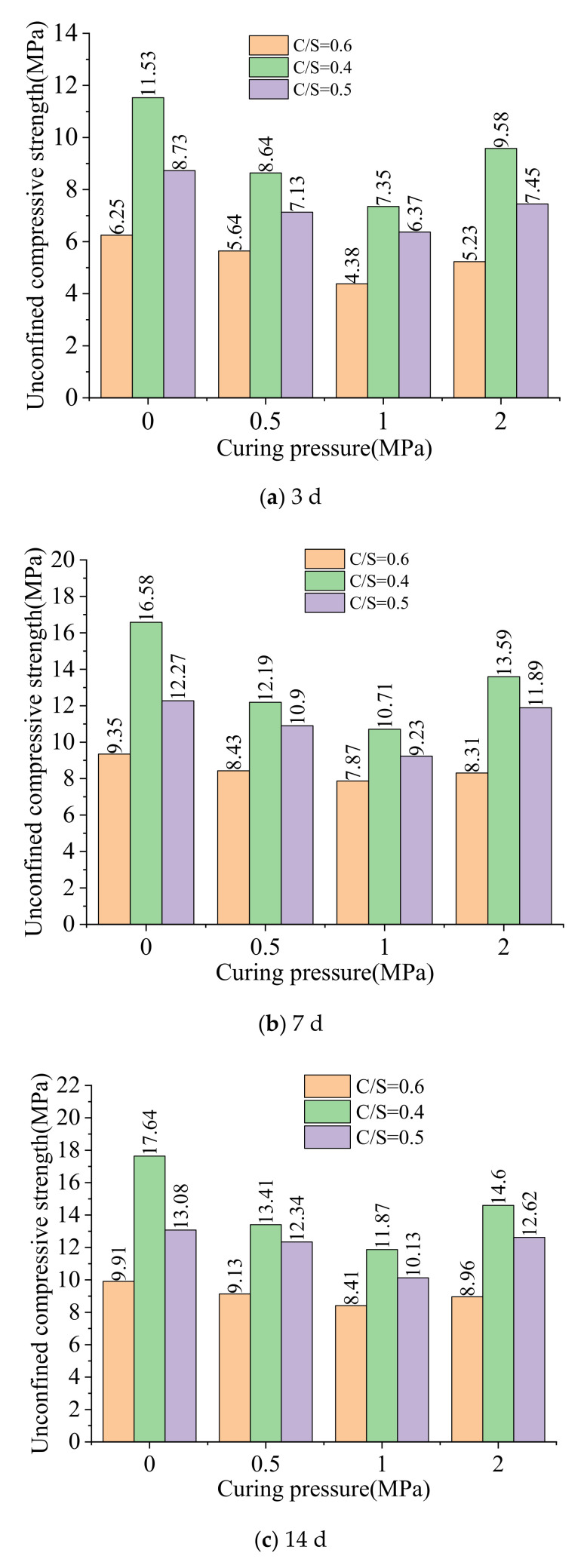
Effect of curing pressure on unconfined compressive strength of hardened grout: (**a**) 3 d; (**b**) 7 d; (**c**) 14 d.

To investigate the effect of curing pressure on the strength of hardened grout, UCS tests were performed on specimens with C/S volume ratios of 0.4, 0.5, and 0.6. The curing pressures were 0 MPa, 0.5 MPa, 1.0 MPa, and 2.0 MPa, and the curing ages were 3 d, 7 d, and 14 d. The results are shown in Figure 11.

In this study, the UCS did not increase monotonically with curing pressure. At all tested curing ages, specimens cured under atmospheric pressure exhibited the highest strength. Strength decreased at 0.5 MPa, reached a minimum at 1.0 MPa, and partially recovered at 2.0 MPa. This non-monotonic trend, characterized by an initial decrease followed by partial recovery, is the key mechanical response observed in the hardened binary grout.

Within the pressure range of 0–1.0 MPa, strength decreased continuously, indicating that curing pressure affected strength not only through pore compaction but also through structural disturbance. Low-to-medium pressure may disrupt the early hydration gel network, resulting in weak interfaces, local defects, and micro-interfacial debonding. When the pressure increased to 2.0 MPa, pore compression, particle interlocking, and structural reconstitution became more effective, thereby partially restoring compressive strength.

In addition, under the same curing pressure and curing age, the strength of the hardened grout decreased as the C/S volume ratio increased. This result confirms that the C/S volume ratio is a key factor controlling the baseline strength of the hardened grout, whereas curing pressure mainly regulates microstructural evolution and the pressure response of the consolidated matrix.

### 3.3. Effect of Water Content on the Properties of Hardened Grout

#### 3.3.1. Effect of Curing Pressure on Water Content

Curing pressure compressed the internal pores of the binary grout and expelled part of the pore water, thereby reducing the water content of the hardened grout. As shown in Table 4, the saturated water content decreased with increasing curing pressure. For C/S = 0.4, the water content decreased from 41.64% at 0 MPa to 39.07% at 2.0 MPa; for C/S = 0.6, it decreased from 44.90% to 41.72%. These results indicate that curing pressure reduced the pore space available for water storage.

At the same curing pressure, specimens with a C/S volume ratio of 0.6 had a higher water content than those with a C/S volume ratio of 0.4. This suggests that increasing the C/S volume ratio increased the internal porosity and water content of the hardened grout. This phenomenon may be related to the abundant gel phases generated by sodium silicate reactions, the relatively loose pore structure, and the higher content of bound and free water. Therefore, the C/S volume ratio is the dominant factor controlling the initial water-bearing state of hardened grout, whereas curing pressure plays a secondary regulating role through compaction.

#### 3.3.2. Cracking and Strength Degradation During Water Loss

Cement–sodium silicate binary grout is characterized by rapid setting and fast early strength development. However, water evaporation from the surface of the consolidated matrix induces shrinkage strain, while internal water migration lags behind and cannot compensate for the volume deformation in time. The resulting differential shrinkage between the exterior and interior generates drying-shrinkage tensile stress. Once this tensile stress exceeds the early tensile strength of the consolidated matrix, primary drying-shrinkage microcracks initiate. The continuous propagation and interconnection of these cracks directly impair the integrity of the hardened grout and significantly reduce its mechanical strength.

**Figure 12:**
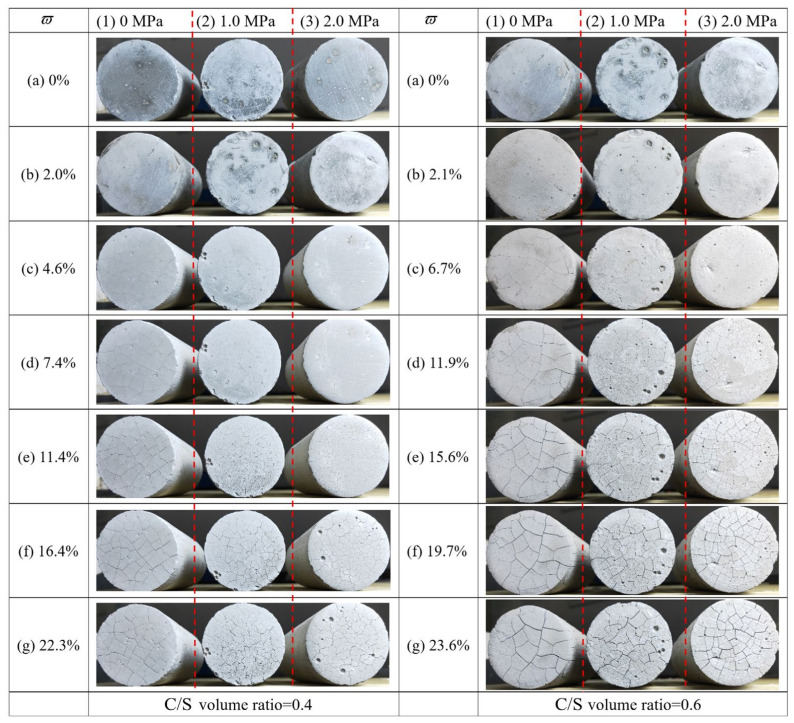
Drying-cracking evolution of specimens with C/S volume ratios of 0.4 and 0.6.

For comparative analysis, the water loss rate, *ϖ*, was defined as the ratio of evaporated water mass to the saturated water mass of the specimen. Labels a–g in Figure 12 represent the surface cracking evolution of specimens with C/S volume ratios of 0.4 and 0.6 cured under different pressures during water evaporation. For the group with a C/S volume ratio of 0.4, the corresponding water loss rates were approximately 0%, 2.0%, 4.6%, 7.4%, 11.4%, 16.4%, and 22.3%. For the group with a C/S volume ratio of 0.6, the corresponding values were approximately 0%, 2.1%, 6.7%, 11.9%, 15.6%, 19.7%, and 23.6%.

As shown in Figure 12, drying-shrinkage cracks evolved stepwise from intact surfaces with micropores to isolated short cracks, partially connected cracks, and finally dense connected crack networks. The water loss rate, curing pressure, and C/S volume ratio jointly controlled the crack-initiation threshold, propagation rate, and final crack morphology.

The water loss rate was the primary internal factor driving crack initiation and propagation. At low water loss rates, matrix shrinkage was limited, and the internal tensile stress did not reach the cracking threshold; therefore, only inherent pores existed on the specimen surface and no visible cracks formed. When the water loss rate exceeded a critical value, scattered short microcracks first appeared at phase interfaces. As the water loss rate further increased, the microcracks propagated along stress-concentration zones and gradually interconnected, forming a network of cracks over the entire specimen surface. A higher water loss rate corresponded to a larger number of wider cracks and smaller matrix fragments separated by cracks.

Increasing the curing pressure effectively restrained the cracking of the hardened binary grout. At the same water loss rate, surface cracks appeared earlier in specimens cured under atmospheric pressure, whereas the critical water loss rate for crack initiation increased with curing pressure. When subjected to the same curing pressure, samples prepared with a C/S volume ratio of 0.6 cracked at a smaller critical water loss rate compared to specimens with a C/S volume ratio of 0.4. Overall, cracking initiated earlier and developed more extensively at C/S = 0.6.

The most severe cracking occurred under the combined condition of C/S = 0.6, water loss rate = 23.6%, and curing pressure = 0 MPa. This indicates that high water loss, zero curing pressure, and a high C/S volume ratio are unfavorable for suppressing drying-shrinkage crack development.

**Figure 13:**
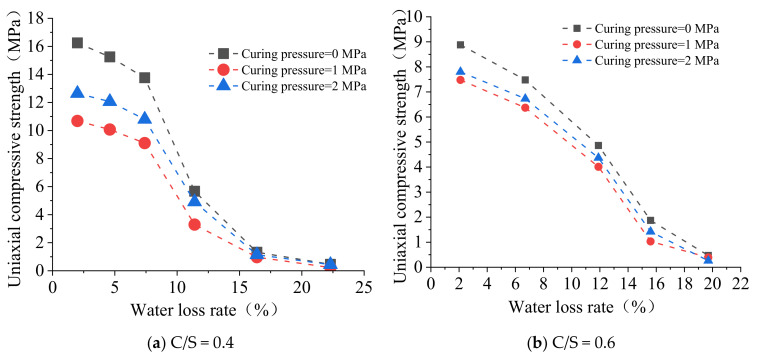
Relationship between water loss rate and compressive strength of specimens with C/S volume ratios of 0.4 and 0.6 under different curing pressures.

Surface cracking during drying was directly accompanied by a significant decrease in compressive strength. As shown in Figure 13, for specimens with the same C/S volume ratio, the strength attenuation trends under different curing pressures were generally consistent as the water loss rate increased.

For specimens with a C/S volume ratio of 0.4, strength degradation can be divided into three stages. When the water loss rate ranged from 0 to 7.4%, compressive strength decreased approximately linearly at a low attenuation rate. When the water loss rate increased from 7.4% to 16.4%, the strength deteriorated rapidly. Once the water loss rate exceeded 16.4%, the strength approached zero, and the matrix essentially lost its load-bearing capacity.

Specimens with a C/S volume ratio of 0.6 exhibited a similar evolution pattern but a faster overall attenuation rate. Their compressive strength decreased sharply when the water loss rate increased from 2.1% to 15.6%, and nearly all mechanical strength was lost at a water loss rate of 19.7%.

## 4. Analysis of Microstructural Mechanisms

### 4.1. SEM Micromorphological Characteristics

**Figure 14:**
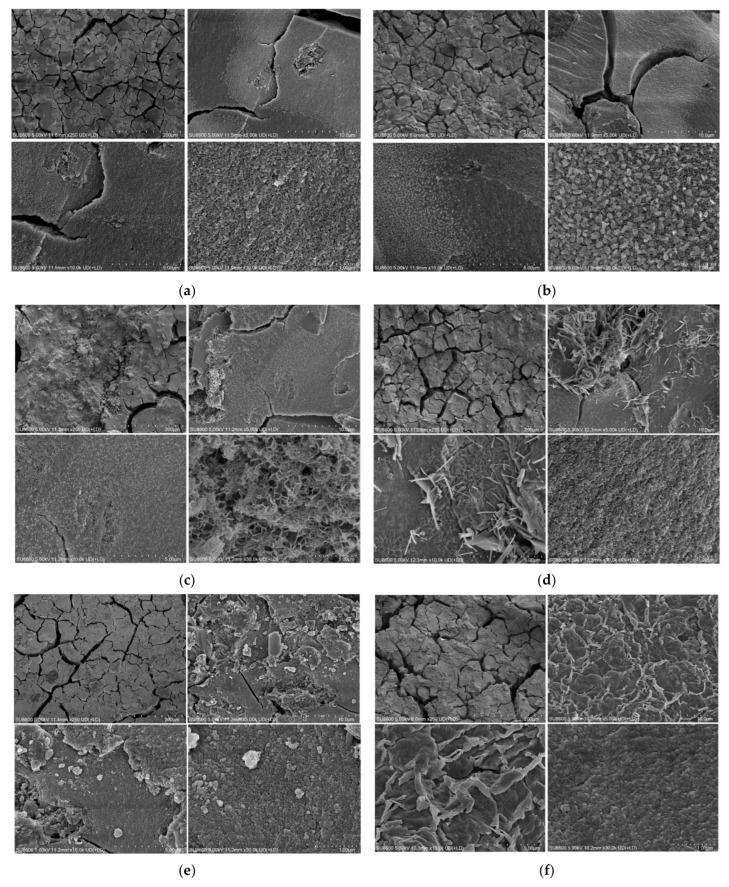
SEM micrographs of hardened grout under different C/S volume ratios and curing pressures: (**a**) C/S = 0.4, 0 MPa; (**b**) C/S = 0.4, 1.0 MPa; (**c**) C/S = 0.4, 2.0 MPa; (**d**) C/S = 0.6, 0 MPa; (**e**) C/S = 0.6, 1.0 MPa; (**f**) C/S = 0.6, 2.0 MPa.

SEM observations revealed that curing pressure markedly modified the microstructure of the hardened binary grout. Specimens with C/S volume ratios of 0.4 and 0.6, cured under different pressures, were observed at four magnification levels, corresponding to fields of view of 200 μm, 10 μm, 5 μm, and 1 μm, as shown in Figure 14. Because microstructural features above the 5 μm scale were insufficient to clearly reflect the pressure-induced evolution mechanism, the following analysis focuses mainly on the 1 μm scale.

For specimens with C/S = 0.4 cured at 0 MPa, continuous and dense hydration gels were observed, with tight solid-phase connections and few internal micropores. Surface cracks were mainly confined to the exterior and did not propagate inward, whereas the internal hydrated skeleton remained intact. At 1.0 MPa, hydration products appeared as discrete particles, and abundant interconnected micron-scale pores were observed between them. This indicates that medium pressure disrupted the continuous hydration gel network and reduced structural integrity. At 2.0 MPa, flocculent calcium silicate hydrate (C-S-H) gels intertwined and filled voids, leaving only a small number of isolated closed micropores. The higher pressure compacted the grout matrix, promoted gel interweaving and densification, and reduced interconnected defects. In this case, compaction and structural reconstitution exceeded the disturbance effect.

For specimens with C/S = 0.6 cured at 0 MPa, hydration products were continuously stacked and tightly bonded. Only scattered, isolated closed pores were present, and no penetrating micron-scale pore channels were observed. At 1.0 MPa, the matrix became loose, and hydration products were distributed as discrete particles separated by numerous interconnected micron-scale pores. This group exhibited the largest pore size, the highest pore connectivity, and obvious interfacial defects. At 2.0 MPa, the matrix became more uniform and compact. Needle-like and flocculent hydration products were compacted and interlocked under high pressure, with only a few independent closed micropores and almost no interconnected voids. This microstructural evolution is consistent with the mechanical trend: the 0 MPa group had the highest strength, the 2.0 MPa group showed partial recovery, and the 1.0 MPa group had the lowest strength.

### 4.2. Porosity and Pore-Size Distribution Characteristics

Mercury intrusion porosimetry (MIP) tests were performed using a fully automatic BELPORE-series instrument manufactured by Microtrac MRB. The instrument includes low-pressure and high-pressure modules; in this study, only the low-pressure module was used, with a maximum pressure of 0.45 MPa. According to the Washburn equation, the intrusion pressure is inversely proportional to the pore diameter. Under the adopted low-pressure condition, detectable pores mainly included macropores and interparticle voids ranging from approximately 1.7 μm to several hundred micrometers. Therefore, the reported porosity values should be interpreted as the pore system detectable by the low-pressure module rather than the complete pore-size range.

**Figure 15:**
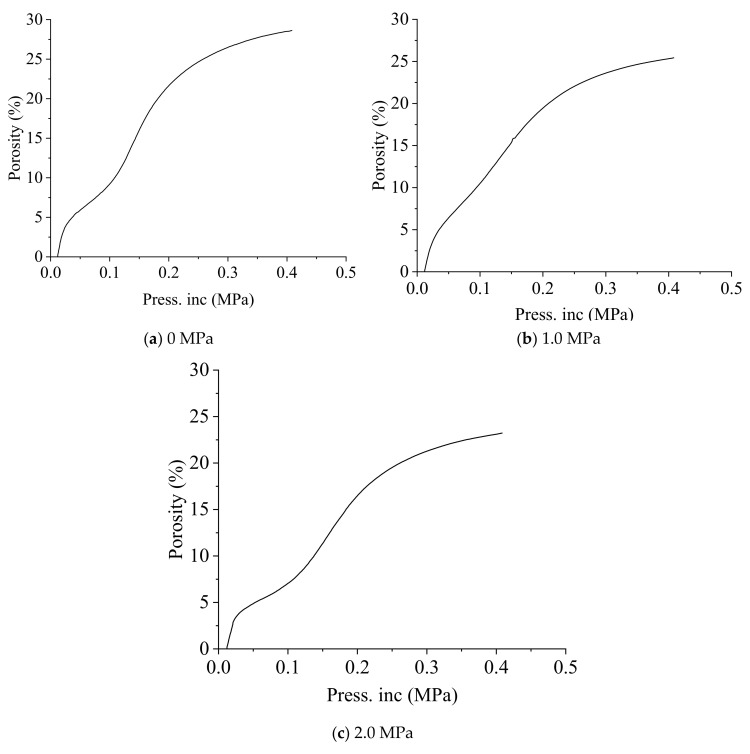
Porosity curves of specimens with C/S = 0.4 under different curing pressures.

**Figure 16:**
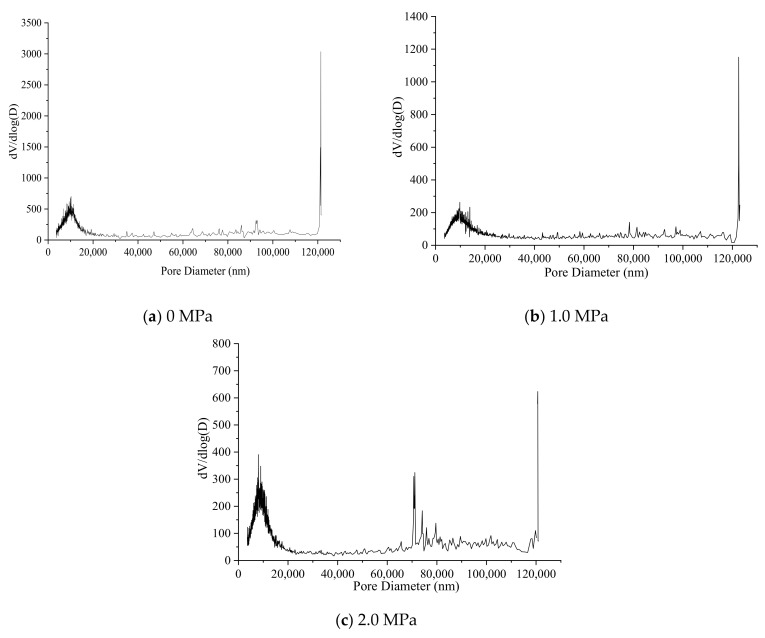
Pore-size distribution curves of specimens with C/S = 0.4 under different curing pressures.

**Figure 17:**
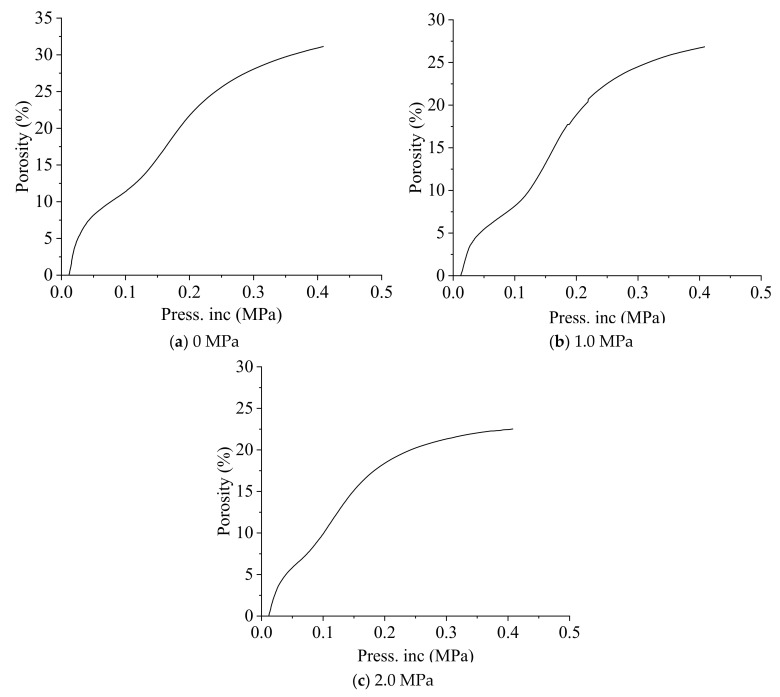
Porosity curves of specimens with C/S = 0.6 under different curing pressures.

**Figure 18:**
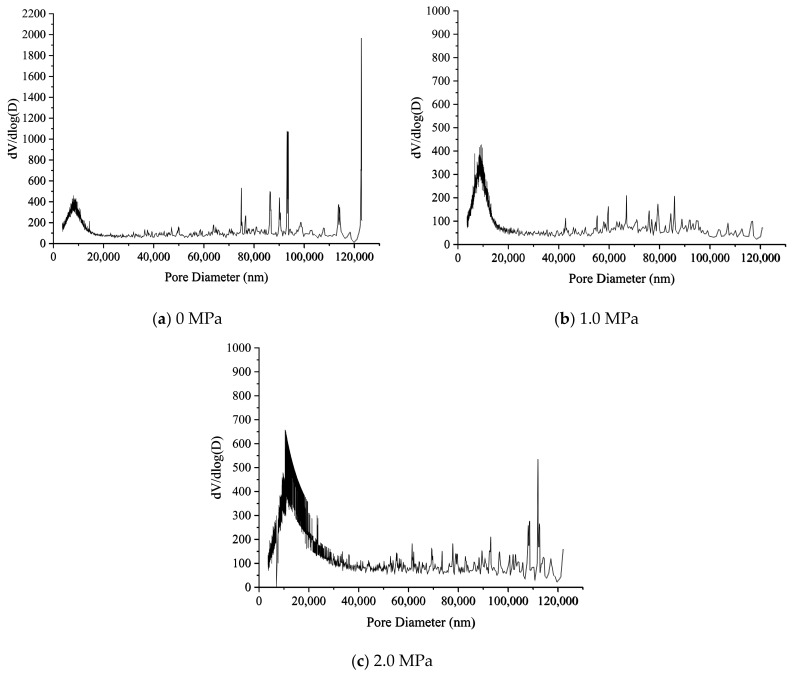
Pore-size distribution curves of specimens with C/S = 0.6 under different curing pressures.

MIP tests were conducted on specimens with C/S volume ratios of 0.4 and 0.6 cured under different pressures, and the results are shown in Figure 15, Figure 16, Figure 17 and Figure 18.

As shown in Figure 15, under a mercury intrusion pressure of 0.45 MPa, the porosities of specimens with C/S = 0.4 cured at 0 MPa, 1.0 MPa, and 2.0 MPa were 28.71%, 25.47%, and 23.76%, respectively. The porosity decreased continuously with increasing curing pressure. All three mercury intrusion curves (0 MPa, 1.0 MPa, 2.0 MPa pre-confining pressures) exhibit identical three-stage variation trends with rising incremental intrusion pressure: porosity rises sharply at low pressure (0–0.1 MPa) as mercury fills large macropores, grows moderately at medium pressure (0.1–0.3 MPa) via mesopore infiltration, and increases slightly to plateau at high pressure (0.3–0.5 MPa) when only tiny micropores remain unfilled. Meanwhile, elevated pre-confining pressure imposes an obvious compaction effect on the material’s pore structure, sequentially lowering the maximum total porosity and instantaneous porosity under the same intrusion pressure, while the hierarchical pore size distribution pattern of the sample remains unchanged.

As shown in Figure 16, specimens with C/S = 0.4 cured at 0 MPa exhibited a typical bimodal pore-size distribution. Ultra-large pores of approximately 120 μm were dominant, followed by small pores in the range of 5–20 μm, whereas intermediate pores were nearly absent. The overall pore volume was large, and the pore-size distribution was highly heterogeneous.

At a curing pressure of 1.0 MPa, both pore peaks decreased substantially. The proportion of ultra-large pores decreased markedly, and the small-pore peak also weakened. The total pore volume was greatly compressed, and the pore-size distribution became flatter.

When the curing pressure increased to 2.0 MPa, the ultra-large pores were further compressed. Meanwhile, a new intermediate-pore peak appeared in the range of 70–80 μm, and the intensity of the small-pore peak increased again. The pore structure therefore exhibited multiple features, including the compression of large pores, the formation of intermediate pores, and the redistribution of small pores, resulting in a more complex pore-size distribution.

As shown in Figure 17, the porosities of specimens with C/S = 0.6 cured at 0 MPa, 1.0 MPa, and 2.0 MPa were 31.24%, 26.73%, and 22.81%, respectively. The porosity decreased gradually as curing pressure increased. The variation law of the above mercury intrusion curves is similar to that under the condition of C/S = 0.4.

As shown in Figure 18, the specimen cured at 0 MPa showed a distinct macropore peak and a high proportion of macropores, corresponding to its relatively loose matrix structure. Under a curing pressure of 1.0 MPa, the macropore peak decreased, the number of macropores was reduced, and the pore-size distribution shifted toward the intermediate-pore range. When the pressure increased to 2.0 MPa, the macropore peak further decreased and became flatter, and the proportion of macropores was further reduced. No obvious secondary pore peak was observed, indicating that the pore structure was continuously optimized without evident high-pressure-induced microdamage. This suggests that the hardened grout with this mix ratio had good structural stability under high-pressure curing and that the compaction effect remained effective throughout the curing process.

Overall, for specimens with C/S = 0.6, the detectable pore volume decreased continuously, macropores were progressively compressed and closed, and the pore structure became denser as the curing pressure increased. This evolution is consistent with the material characteristics of relatively high plasticity and low baseline compressive strength.

A comparison of the two groups reveals clear differences in the pore evolution of hardened binary grout under curing pressure, particularly in their high-pressure responses. Specimens with C/S = 0.4 exhibited higher strength but poorer plasticity. Partial densification occurred under pressure; however, a high pressure of 2.0 MPa might induce internal microcracks or pore redistribution. In contrast, specimens with C/S = 0.6 exhibited better plasticity, lower strength, and higher pressure sensitivity. Their macropores were more readily compressed and closed under pressure, producing a more pronounced compaction effect.

These results indicate that the pressure-dependent pore evolution of hardened binary grout is strongly governed by the C/S volume ratio. Increasing the C/S volume ratio improves structural adaptability under high-pressure curing but reduces the baseline compressive strength. The reduction in total porosity fails to induce a corresponding increase in mechanical strength, which can be attributed to the transformation of the pore structure rather than the change in total pore volume alone. With the increase in curing pressure, interconnected macropores are compressed and closed, leading to a decline in overall porosity. However, the compaction process generates a large number of tiny isolated micropores and internal microcracks inside the hardened grout matrix. These newly formed fine pores and microfractures act as internal stress concentration zones under external load. Although the total void volume decreases, the abundant micro-defects serve as crack initiation sites. The adverse weakening effect of widespread microcracks offsets the strengthening effect brought by reduced porosity, thus resulting in the phenomenon where strength does not rise despite lower total porosity.

## 5. Conclusions

(1) Increasing the bentonite content significantly reduced the bleeding rate of the cement slurry, and an optimal superplasticizer dosage minimized bleeding. Under the present test conditions, 3% bentonite combined with 1% polycarboxylate superplasticizer effectively improved slurry stability. Increasing the C/S volume ratio enhanced fluidity and prolonged gel time; considering both workability and hardened mechanical performance, C/S volume ratios of 0.4, 0.5, and 0.6 were selected for compressive-strength tests.

(2) Under atmospheric curing pressure, the UCS of hardened binary grout increased with curing age, and strength gain mainly occurred within the first 7 days. At the same curing age, compressive strength decreased as the C/S volume ratio increased, following the order C/S = 0.4 > C/S = 0.5 > C/S = 0.6; Curing pressure exerted a non-monotonic effect on compressive strength. UCS decreased as curing pressure increased from 0 to 1.0 MPa and then partially recovered at 2.0 MPa. Pressurized curing duration strongly affected early-age strength, but this effect weakened after 3 d.

(3) The compressive strength of hardened binary grout continuously decays as its water content decreases, accompanied by the gradual surface cracking of the matrix. The attenuation law of compressive strength with a rising water loss rate can be divided into three successive stages: a slight approximate linear reduction in strength, rapid and severe strength deterioration, and complete loss of bearing capacity. Meanwhile, grout with a C/S volume ratio of 0.6 possesses a lower critical water loss rate for surface cracking compared with specimens with a C/S volume ratio of 0.4.

(4) SEM and MIP results showed that curing pressure induced both pore compaction and structural disturbance. At 1.0 MPa, particle dispersion, weak interfacial bonding, and discontinuous microstructure explained the strength minimum. At 2.0 MPa, enhanced compaction, structural reconstitution, and particle interlocking partially restored compressive strength. Grout with a higher C/S volume ratio exhibited better plasticity and pressure adaptability but lower baseline strength.

This study only carried out indoor laboratory tests with limited discrete curing pressure gradients, and merely conducted qualitative SEM microscopic observation without quantitative statistical analysis of pore characteristic parameters due to exhausted test specimens; meanwhile, field construction verification and long-term dry–wet cycle durability tests under complex underground service environments were not performed. In addition, the specific evolution law and microscopic mechanism regarding how curing pressure modifies the internal structure of hardened binary grout remain to be further explored. Subsequent research will adopt denser pressure gradients to identify the critical strength transition pressure, supplement quantitative pore structure characterization, verify the optimized mix proportion under actual on-site working conditions, and implement dry–wet cycle tests to evaluate the long-term mechanical stability of pressurized-cured cement-sodium silicate binary grout.

## Figures and Tables

**Table 1:** Bleeding rate test results (unit: %).

Superplasticizer Dosage (%)	Bentonite Dosage (%)			
	0	1	2	3
0.5	28.13	19.6	12.42	8.66
1	26.23	15.61	8.83	4.78
1.3	29.42	22.34	17.87	13.86

**Table 2:** Experimental results of fluidity of double slurries under three kinds of sodium silicate modulus (Unit: mm).

Sodium Silicate Concentrations (°Bé)	C/S Volume Ratio			
	0.4	0.5	0.6	0.7
39	289.2	312.7	318.4	317.9
36	298.1	315.6	320.8	321.9
32	301.9	318.4	322.3	323.4

**Table 3:** The gel time test results with different C/S volume ratios and sodium silicate concentrations (Unit: s).

Sodium Silicate Concentrations (°Bé)	C/S Volume Ratio					
	0.3	0.4	0.5	0.6	0.7	0.3
39	28	40	56	83	103	39
32	36	49	66	96	117	32

**Table 4:** Water content of hardened binary grout under different curing pressures.

C/S volume ratio	0.4	0.4	0.4	0.6	0.6	0.6
Curing pressure	0 MPa	1.0 MPa	2.0 MPa	0 MPa	1.0 MPa	2.0 MPa
Water content (%)	41.64	40.31	39.07	44.90	42.61	41.72

## Data Availability

The original contributions presented in this study are included in the article. Further inquiries can be directed to the corresponding author.
